# Unveiling the Diagnostic Potential: A Comprehensive Review of Bronchoalveolar Lavage in Interstitial Lung Disease

**DOI:** 10.7759/cureus.52793

**Published:** 2024-01-23

**Authors:** Arman Sindhu, Ulhas Jadhav, Babaji Ghewade, Pankaj Wagh, Pallavi Yadav

**Affiliations:** 1 Respiratory Medicine, Jawaharlal Nehru Medical College, Wardha, IND; 2 Obstetrics and Gynecology, Jawaharlal Nehru Medical College, Wardha, IND

**Keywords:** biomarker discovery, multidisciplinary approach, idiopathic pulmonary fibrosis, diagnostic potential, interstitial lung disease, bronchoalveolar lavage

## Abstract

This comprehensive review examines the diagnostic potential of bronchoalveolar lavage (BAL) in interstitial lung disease (ILD), emphasizing its accuracy and significance in various ILDs, including idiopathic pulmonary fibrosis (IPF), sarcoidosis, hypersensitivity pneumonitis, and connective tissue disease-associated ILD. The analysis underscores the importance of abnormalities in both cellular and non-cellular components of BAL fluid for precise ILD diagnosis. Recommendations advocate for the integration of BAL into clinical guidelines, a multidisciplinary diagnostic approach, and further standardization of procedures. Looking toward the future, ongoing research highlights technological advancements, biomarker discovery, and the integration of artificial intelligence in BAL interpretation. These developments not only promise to enhance ILD diagnosis but also offer prospects for a more personalized approach to patient management based on insightful patient stratification guided by BAL findings. This abstract encapsulates the key findings, recommendations, and future prospects identified in the review, providing a concise overview of the diagnostic potential of BAL in ILD.

## Introduction and background

Interstitial lung disease (ILD) represents a diverse group of disorders characterized by inflammation and fibrosis within the lung parenchyma. These conditions collectively pose a significant burden on global health, contributing to morbidity and mortality. Understanding the pathophysiology and diagnosing ILD present considerable challenges, necessitating advanced diagnostic approaches. Among these, bronchoalveolar lavage (BAL) has emerged as a valuable tool in unraveling the complexities of ILD [1]. ILD refers to a group of diffuse parenchymal lung disorders, encompassing a spectrum of conditions such as idiopathic pulmonary fibrosis (IPF), sarcoidosis, and connective tissue disease-associated ILD (CTD-ILD). Characterized by inflammation and fibrosis of the interstitium, ILD results in impaired lung function and, in severe cases, respiratory failure. The significance of ILD lies in its impact on patients’ quality of life, the challenge it poses to healthcare systems, and the need for accurate and timely diagnosis for appropriate management [2].

Diagnosing ILD is a complex task due to the heterogeneous nature of these disorders. The absence of specific clinical symptoms and the overlap with other respiratory conditions often lead to delayed or misdiagnosed cases. Distinguishing between different ILD subtypes and identifying disease progression present ongoing challenges in clinical practice. Moreover, the invasive nature of some diagnostic procedures raises concerns about patient comfort and safety [3]. Amid the diagnostic challenges in ILD, BAL emerges as a key investigative tool. BAL involves the instillation and subsequent retrieval of a sterile saline solution within a specific lung segment, allowing for the collection of bronchoalveolar fluid. This fluid, rich in cells and soluble mediators, serves as a window into the pulmonary microenvironment. The cellular and molecular composition of BAL fluid provides valuable insights into the underlying pathology, aiding in the differential diagnosis of ILD subtypes [4].

The purpose of this review is to comprehensively explore the diagnostic potential of BAL in ILD. By delving into the historical context, procedural nuances, and clinical applications of BAL, we aim to elucidate its role in enhancing the accuracy and efficiency of ILD diagnosis. Furthermore, we will discuss the current state of knowledge, methodological advancements, and potential future directions, shedding light on the evolving landscape of ILD diagnostics.

## Review

Background of BAL

Definition and Purpose of BAL

BAL is a minimally invasive procedure conducted during flexible bronchoscopy to acquire a sample of alveolar cells [5]. The process entails introducing sterile normal saline into a specific lung segment, followed by the retrieval and analysis of the instilled fluid [6]. In the diagnostic assessment of ILD, BAL assumes a crucial role by furnishing valuable insights into alveolar cell patterns. When coupled with comprehensive clinical information and high-resolution computed tomography (HRCT), BAL aids in achieving a precise and confident diagnosis [7]. This procedure is deemed low risk and is routinely employed for both diagnostic and research purposes [6]. Moreover, it serves to identify complicating factors such as infections or malignancies [7]. The decision to undertake BAL should be guided by the patient’s clinical presentation, with the interpretation of findings entrusted to experts in the context of the clinical situation [6]. Despite BAL’s widespread acceptance as a valuable diagnostic tool, ongoing efforts toward standardizing techniques and protocols for processing and analyzing BAL fluid persist. Unresolved questions include determining the optimal technique for conducting BAL [7].

Historical Context and Development of BAL in ILD Diagnosis

The inception of BAL as a diagnostic tool for ILD can be traced back to the 1980s. Since its introduction, BAL has played a crucial role in obtaining alveolar cell samples, aiding in the diagnosis and monitoring of ILD patients [1]. The procedure involves the infusion of sterile normal saline into a specific lung segment, followed by the retrieval and analysis of the instilled fluid [8].

BAL has found application in various ILDs over the years, including hypersensitivity pneumonitis (HP), CTD-ILD, and sarcoidosis [8]. Notably, extreme lymphocytosis in BAL is considered indicative of HP, while pronounced lymphocytosis (≥40%) is associated with CTD-ILD, sarcoidosis, and HP [8]. However, it is essential to acknowledge that neither the presence nor the degree of BAL lymphocytosis can definitively point to a specific diagnosis [8].

The diagnostic value of BAL in ILD management has been a subject of continuous debate and controversy [9]. While BAL is acknowledged as a valuable diagnostic tool, its role in routine clinical management remains a topic of discussion [9]. Studies have sought to evaluate BAL’s diagnostic value in ILD management by comparing cytological findings in BAL fluid across different diseases within the ILD spectrum [10]. Nevertheless, the ongoing standardization of techniques and protocols for processing and analyzing BAL fluid underscores the need for further research and consensus in this area [9]. In summary, while BAL has been instrumental in ILD diagnosis since the 1980s, its role in routine clinical management and the ongoing standardization of techniques for processing and analyzing BAL fluid continue to be subjects of ongoing debate and research.

BAL as a Minimally Invasive Procedure

BAL is a minimally invasive procedure conducted during flexible bronchoscopy aimed at acquiring alveolar cell samples [4,5]. This entails the introduction of sterile normal saline into a specific lung segment, followed by the retrieval and subsequent analysis of the instilled fluid [4]. Recognized as a low-risk diagnostic tool, BAL is routinely employed in the assessment of ILD to obtain crucial diagnostic information [4]. The procedure involves the insertion of a flexible bronchoscope into a subsegment of the lung [6]. Notably, BAL serves a dual purpose, being both diagnostic and therapeutic, while also proving instrumental as a research tool for ILD. It offers valuable insights into the composition of immune effector cells accumulating in the alveoli [4]. The decision to employ BAL should be informed by the patient’s clinical presentation, and the interpretation of its findings should be entrusted to experts who can contextualize the results within the broader clinical situation [4].

Techniques of BAL

Procedure and Equipment Used in BAL

BAL is a minimally invasive procedure conducted during flexible bronchoscopy with the primary objective of obtaining a sample of alveolar cells [5]. The technique involves the careful instillation of sterile normal saline into a specific subsegment of the lung, followed by the meticulous suction and collection of the instilled fluid for subsequent analysis [6]. Typically, BAL is performed during bronchoscopy under local anesthesia and moderate sedation, although the ideal conditions, albeit less common, involve general anesthesia and a rigid bronchoscope [4]. Guidelines recommend a minimum lavage fluid volume of around 100 mL for adults, with recommendations extending from 100 to 300 mL for BAL [4].

The essential equipment used in BAL includes a bronchoscope, sterile normal saline solution, a suction catheter, and a collection container [11]. The bronchoscope, characterized by its thin and flexible tube equipped with a light and camera, is inserted through the mouth or nose and navigated down the throat into the airways [11]. The suction catheter plays a crucial role in aspirating the instilled saline solution and facilitating the collection of the BAL fluid [11]. This comprehensive setup ensures the procedural success of BAL, enabling the retrieval of diagnostic samples while maintaining patient comfort during the bronchoscopic examination.

Collection and Processing of BAL Fluid

Maintaining the integrity and viability of BAL fluid is imperative for accurate diagnostic analysis, prompting the need for prompt processing. Experts recommend storing BAL fluid samples at 4°C before processing to ensure optimal preservation of sample quality and cell viability. Time sensitivity in processing is underscored to prevent any compromise in sample integrity, and it is essential to appropriately label and handle BAL fluid samples with precision [12]. During transport to the laboratory, maintaining the viability of cells is achieved by storing the BAL fluid on ice, with research indicating that cells in BAL fluid can remain viable for up to four hours at room temperature.

The fluid analysis typically involves assessing white and red cells, employing various staining techniques to evaluate the presence of different cell types and microorganisms [6]. In specific instances, filtration of BAL samples may be deemed necessary before processing to eliminate contaminants like mucus plugs. However, careful consideration is crucial when contemplating filtration, as it has the potential to impact the diagnostic quality of the sample by removing microorganisms and cells that may carry valuable diagnostic information [12]. In essence, the collection and processing of BAL fluid necessitate adherence to specific storage conditions, meticulous transportation on ice, and a thorough analysis of various cell types and microorganisms using staining techniques.

Standardization of BAL Techniques for ILD Diagnosis

The standardization of BAL techniques for diagnosing ILD remains an active focus of both research and clinical guideline development. While BAL is acknowledged as a valuable tool in the diagnostic assessment of ILD patients, its precise diagnostic value in differentiating between the diverse entities within the heterogeneous ILD group remains a subject of ongoing controversy [10]. The aspiration is that evolving guidelines will enhance the utility of BAL in the diagnostic evaluation of ILD and encourage its incorporation into clinical studies [9]. When utilized in conjunction with comprehensive clinical information and HRCT, BAL proves instrumental in offering valuable insights for diagnostic purposes, including the identification of confounding conditions such as infection or malignancy [7]. The critical standardization of BAL techniques and protocols for processing and analyzing BAL fluid becomes paramount for furnishing valuable diagnostic information in ILD cases [7].

Although BAL is widely accepted as a relatively safe and straightforward diagnostic procedure, ongoing efforts toward technique standardization and controlled clinical trials are imperative to determine its efficacy and durability. Accurate interpretation of BAL cellular analyses in ILD necessitates comprehensive clinical information, and the differential diagnosis of these disorders relies on the clinician’s astute interpretation of patient data, amalgamated with various diagnostic measures, including BAL [13]. The quest for standardized BAL techniques and protocols, coupled with ongoing clinical trials, reflects the commitment to optimizing the diagnostic potential of BAL in the complex landscape of ILD.

Indications for BAL in ILD

Clinical Scenarios Where BAL Is Particularly Useful

Discriminating between ILD entities: BAL plays a pivotal role in enhancing the accuracy of ILD diagnosis by aiding in the differentiation of specific ILD forms. Notably, it contributes to the precise identification of entities such as idiopathic interstitial pneumonias, granulomatous lung diseases, and other miscellaneous ILDs [7]. This capability is particularly significant in tailoring treatment strategies and prognostic assessments for patients with distinct ILD subtypes.

Diagnosing inflammatory and infectious processes: The cellular analysis of BAL is invaluable for diagnosing inflammatory and infectious processes occurring at the alveolar level among ILD patients. By scrutinizing cellular components in the BAL fluid, clinicians can gain crucial insights into the underlying pathogenic mechanisms, allowing for a more targeted and effective approach to managing ILD patients experiencing inflammatory or infectious complications [9]. This diagnostic capability is integral to comprehensive ILD patient care.

Assessing treatment response: BAL serves as a valuable tool in assessing the response to treatment among ILD patients, particularly those undergoing complex interventions such as stem cell or organ transplantation. Monitoring changes in cellular composition and other relevant markers in the BAL fluid enables clinicians to gauge the effectiveness of therapeutic interventions, guide treatment adjustments, and optimize patient outcomes [7]. This aspect underscores BAL’s role not only in diagnosis but also in the ongoing management and treatment optimization in the context of ILD.

Excluding other conditions: BAL is instrumental in the diagnostic workup of ILD by assisting in the exclusion of various conditions that may mimic or coexist with ILD. Conditions such as cryptogenic organizing pneumonia, CTDs, extrinsic allergic alveolitis, and respiratory bronchiolitis-associated ILD can be effectively ruled out through BAL analysis [7]. This exclusionary function enhances diagnostic precision and ensures a more focused and tailored approach to ILD management.

Situations Where BAL May Not Be the First-Line Diagnostic Tool

Clear diagnosis: In instances where the diagnosis of ILD is evident based on robust clinical and radiological findings, the use of BAL may be deemed unnecessary [6]. When the clinical presentation and imaging results provide a clear and conclusive diagnosis, incorporating BAL into the diagnostic workup might not offer additional substantive insights. This selective approach ensures that diagnostic procedures are employed judiciously, avoiding unnecessary interventions when a definitive diagnosis has already been established through other means.

Risk-benefit imbalance: There are situations where the potential risks associated with performing BAL may outweigh the anticipated benefits, particularly in patients with severe hypoxemia or hemodynamic instability [5]. In these cases, the decision to use BAL is carefully evaluated, considering the heightened risks and potential complications associated with the procedure. Balancing the potential benefits of obtaining diagnostic information through BAL against the risks is crucial to ensure patient safety and well-being.

Appropriateness of other diagnostic tools: Depending on the specific clinical context, alternative diagnostic tools, such as transbronchial lung biopsy or surgical lung biopsy, may be more suitable than BAL [7]. The choice of diagnostic modality is guided by factors such as the nature of the suspected ILD, the level of diagnostic uncertainty, and the overall clinical condition of the patient. This tailored approach ensures that the most appropriate and effective diagnostic tools are employed based on the unique characteristics of each case.

Patient tolerance: The feasibility of performing BAL is contingent on the patient’s ability to tolerate the procedure. If a patient is unable to undergo BAL due to comorbidities or other factors, alternative diagnostic strategies may be considered [14]. Patient comfort, safety, and overall well-being are paramount considerations, and the decision to proceed with BAL is made in recognition of the patient’s ability to undergo the procedure without undue distress or risk. While BAL is acknowledged as a useful diagnostic tool in ILD, it may not always be the initial or first-line diagnostic choice. The decision to perform BAL is highly individualized, taking into account the specific clinical presentation, associated risks, and potential benefits for each patient [5-7,14]. This personalized and case-by-case approach ensures that diagnostic strategies align closely with the unique circumstances of each ILD case, optimizing the balance between diagnostic yield and patient safety.

Comparison of BAL With Other Diagnostic Methods in ILD

BAL proves to be a valuable tool in the diagnostic evaluation of individuals with ILDs. Multiple studies have demonstrated the utility of BAL in providing pertinent diagnostic information, particularly when utilized alongside comprehensive clinical data and HRCT. The diagnostic yields of BAL among ILD patients were found to be 60.6%, 69.7%, and 21.6% in ICU, general ward, and outpatient department patients, respectively [13]. Despite its recognized diagnostic benefits, the use of BAL in distinguishing between the diverse entities within the heterogeneous ILD group remains a subject of controversy [10]. While BAL can significantly contribute to achieving an accurate and confident diagnosis of specific ILD forms, it is not recommended for use in cases of non-specific interstitial pneumonia and desquamative interstitial pneumonia [7]. Combining clinical information and HRCT findings with the analysis of BAL fluid enhances the likelihood of arriving at confident diagnoses, potentially eliminating the necessity for a surgical lung biopsy [7]. The cellular analysis of BAL fluid emerges as a valuable diagnostic aid, particularly in cases of undifferentiated ILD [15].

Components of BAL fluid

Cellular Components

Macrophages: Constituting over 80% of the cells in BAL fluid, macrophages are the predominant cell type. Playing a pivotal role in the immune response, these cells are crucial for clearing pathogens and debris within the lung [4,16]. Their prevalence underscores their significance in maintaining pulmonary health and responding to potential threats.

Lymphocytes: Accounting for 5-15% of the BAL fluid population, lymphocytes are vital components of the immune system. Their presence in BAL fluid serves as an essential aspect for discerning between various forms of ILD [4,16]. The differential composition of lymphocytes contributes valuable information to the diagnostic process, aiding in the characterization of specific ILD entities.

Neutrophils: Comprising 1-3% of the BAL fluid population, neutrophils, a type of granulocyte, hold a notable role in the immune response, particularly in the context of infections [10]. Their presence in BAL fluid can provide insights into the inflammatory status of the lung and contribute to the diagnostic evaluation, especially when infections are suspected.

Eosinophils: Making up 1% of the BAL fluid population, eosinophils participate in the immune response, especially in reaction to parasitic infections [17]. Their presence in BAL fluid can serve as an indicator of specific immune responses, aiding in the identification and understanding of underlying pathological processes, particularly those associated with parasitic infections.

Mast cells: Present in trace amounts in BAL fluid, mast cells contribute to the immune response, particularly in reaction to allergens and certain medications [4]. Although their prevalence is minimal, their involvement in immune reactions highlights their potential significance in specific contexts, such as allergic responses. The relative proportions of these distinct cell types can vary based on the individual’s health status and the specific form of ILD under consideration. It is imperative to interpret BAL findings within the broader context of clinical information and other diagnostic tools. This comprehensive approach ensures the accurate diagnosis and effective monitoring of ILD, recognizing the dynamic interplay between cellular components and the clinical landscape [4,17].

Non-Cellular Components

Cytokines: Essential proteins in the immune response and inflammation, cytokines, including interleukins, tumor necrosis factor (TNF), and interferons, can be quantified in BAL fluid to evaluate the inflammatory status of the lung [18,19]. These signaling molecules serve as key regulators of immune processes, and their measurement in BAL fluid provides valuable insights into the dynamic immunological environment within the lung.

Growth factors: Proteins that facilitate cell growth and differentiation, growth factors, such as platelet-derived growth factor and epidermal growth factor, are present in BAL fluid and offer potential indications of the lung’s overall health status [18,19]. Their presence and levels in BAL fluid contribute to understanding the cellular dynamics and regenerative processes within the lung microenvironment.

Chemokines: Functioning as proteins that attract and guide leukocytes to inflammatory sites, chemokines like eotaxin, monocyte chemoattractant protein-1, and interferon-induced inducible protein-10 can be assessed in BAL fluid to gauge the inflammatory response in the lung [18,19]. The measurement of chemokines provides valuable information on the recruitment and activation of immune cells in response to inflammation.

Cathepsins: Enzymes involved in protein degradation, cathepsins identified in BAL fluid offer insights into proteolytic activity within the lung and may be associated with specific lung diseases [18,19]. Monitoring cathepsin levels provides information about the breakdown of proteins, contributing to a more comprehensive understanding of pathological processes in lung diseases.

Oxidative stress markers: Including reactive oxygen species and advanced glycation end products, oxidative stress markers measurable in BAL fluid serve as indicators of the oxidative stress status of the lung [18,19]. The assessment of these markers sheds light on the balance between oxidants and antioxidants, providing crucial information about the potential impact of oxidative stress on lung health. The analysis of non-cellular components in BAL fluid employs various techniques, such as enzyme-linked immunosorbent assays, Western blotting, and multiplex bead-based immunoassays. By measuring these components, researchers gain a deeper understanding of the underlying mechanisms and responses in lung diseases. This knowledge, in turn, contributes to the development of targeted therapies and diagnostic tools aimed at addressing specific molecular and biochemical aspects of lung pathophysiology [17-19].

Significance of Abnormalities in BAL Fluid Components in ILD

The analysis of BAL fluid stands as a valuable resource in the assessment of ILD. Specific characteristics within BAL fluid, such as the CD4+/CD8+ lymphocyte ratio, can furnish distinctive diagnostic insights in certain atypical ILDs, aiding in the differentiation of conditions like HP, sarcoidosis, and pulmonary alveolar proteinosis [20]. However, the diagnostic utility of BAL fluid parameters, encompassing lymphocyte count and CD4/CD8 ratio, lacks specificity and diagnostic precision for any ILDs due to notable variability in these values [20].

In individuals without ILD, BAL fluid predominantly comprises alveolar macrophages, with a minor presence of lymphocytes, neutrophils, or eosinophils [20]. In ILD patients, differential cell counts and specific BAL lavage features exhibit variability, non-specificity, and limited sensitivity. Nevertheless, the analysis of BAL cellular profiles holds the potential for refining the differential diagnoses of ILDs and guiding further evaluation [4]. Furthermore, cellular analysis of BAL fluid is under consideration as a potential biomarker for predicting prognosis in acute exacerbations of ILD [21].

Despite being a non-invasive diagnostic procedure for ILDs, the discerning power of BAL in distinguishing between entities within the diverse ILD group remains a topic of controversy [10]. Consequently, the interpretation of BAL fluid components necessitates consideration within the broader context of clinical and radiological findings to realize its significance in ILD evaluation [7].

Diagnostic value of BAL in specific ILDs

IPF

IPF is the most prevalent form of pulmonary fibrosis, a condition characterized by the development of scarring and rigidity in the lungs, leading to increased difficulty in breathing [22,23]. The precise cause of IPF remains elusive, and while its onset typically occurs in individuals aged 70-75, it is relatively rare in those under 50 [22]. Several potential risk factors for IPF include exposure to specific types of dust, viral infections, a familial history of IPF, acid reflux, and smoking [22]. However, the direct causative relationship between these factors and IPF remains unclear. Symptoms of IPF manifest gradually, worsening over time as the tiny air sacs in the lungs, known as alveoli, undergo damage and progressive scarring, resulting in increased lung stiffness and hindering the transfer of oxygen into the bloodstream [22]. The term “idiopathic” is employed because the precise cause of this scarring process remains unknown [23].

The diagnosis of IPF poses challenges due to its symptom overlap with other lung diseases [23]. Healthcare professionals employ a combination of diagnostic approaches, including auscultation with a stethoscope to assess lung sounds, detailed inquiries about the patient’s symptoms, and a battery of tests such as lung function assessments, blood tests, chest X-rays, CT scans, and potentially a lung biopsy [23,24]. Crucially, a definitive diagnosis of IPF is contingent upon the inability to identify an underlying cause and the presence of a discernible pattern of scarring in the lungs [25]. This comprehensive diagnostic process ensures a thorough evaluation and accurate determination of IPF, facilitating appropriate management and care for affected individuals.

Sarcoidosis

BAL emerges as a valuable diagnostic tool for identifying sarcoidosis, a specific subtype of ILD. According to a study, the likelihood of sarcoidosis significantly increased from 33.7% to 68.1% when lymphocyte numbers were elevated in BAL fluid [10]. Another research endeavor indicated that detecting 1% or less neutrophils in BAL had an 80% positive predictive value in distinguishing sarcoidosis from ILDs unrelated to sarcoidosis [26]. Despite these promising findings, the diagnostic utility of BAL in effectively differentiating among the diverse entities within the heterogeneous ILD spectrum remains a contentious matter. Ongoing debates persist, and efforts toward standardizing techniques and conducting controlled clinical trials continue to ascertain the efficacy and reliability of BAL in this diagnostic context [10,13].

HP

HP is an ILD characterized by inflammation and/or fibrosis triggered by exposure to an antigen. BAL, a minimally invasive procedure, holds the potential to furnish valuable diagnostic information for specific ILDs, including HP. Nevertheless, the diagnostic value of BAL in the context of HP remains a subject of controversy. A systematic review conducted by Tzilas et al. indicated that a BAL lymphocytosis exceeding 20% demonstrated added diagnostic value in a retrospective cohort of undiagnosed fibrotic ILD [8]. However, conflicting conclusions regarding the diagnostic utility of BAL cellular analysis in ILD have been reported by other studies [10].

In specific cases, such as distinguishing IPF from alternative conditions like eosinophilic pneumonia, cryptogenic organizing pneumonia, sarcoidosis, or infections, BAL findings exhibit high specificity and diagnostic accuracy [27]. However, in more prevalent ILDs, BAL findings lack specificity and cannot independently yield a confident diagnosis. When considered alongside clinical information and HRCT, the analysis of BAL cellular components may aid in narrowing the differential diagnosis and potentially obviating the need for a surgical lung biopsy [27].

The diagnostic role of BAL in HP remains contentious, with some studies proposing that BAL lymphocytosis can enhance diagnostic accuracy in certain instances, while others present contradictory results. Further research is imperative to conclusively establish the role of BAL in diagnosing HP and other ILDs, contributing to a more comprehensive understanding of its diagnostic potential in different clinical contexts.

CTD-ILD

The diagnostic utility of BAL, in particular for ILDs such as CTD-ILD, remains a subject of continuous research and deliberation. BAL is acknowledged as a valuable tool in the diagnostic assessment of ILD patients, offering significant information when integrated with clinical data and imaging. However, its precise diagnostic efficacy in distinguishing among the diverse entities within the heterogeneous ILD spectrum remains a topic of ongoing debate. A retrospective study aimed at assessing BAL’s diagnostic value in hospitalized ILD patients revealed that the cellular profile derived from BAL, in conjunction with medical history, physical examination, and imaging findings, may either support or help narrow down the likely diagnostic hypothesis [28,29]. Correspondingly, the American Thoracic Society underscores that the accurate interpretation of BAL cellular analyses in ILD necessitates comprehensive clinical information, with the differential diagnosis relying on the clinician’s integration of patient details and various diagnostic measures, including BAL [9].

While BAL is widely acknowledged as a straightforward and relatively safe diagnostic procedure, efforts to standardize techniques and conduct controlled clinical trials to determine its efficacy and durability are still ongoing. Consequently, despite its recognized utility in the diagnostic evaluation of ILD patients, the specific diagnostic value of BAL in CTD-ILD and other distinct ILDs remains a subject of continual research and clinical discourse [10,13,28].

Limitations and challenges of BAL

False Positives and Negatives

BAL proves to be a valuable tool with both strengths and limitations in the diagnosis of ILD. Its significant role in achieving an accurate and confident diagnosis, especially when used in conjunction with comprehensive clinical information and HRCT, is notable. However, it is essential to consider its limitations. A systematic review and meta-analysis indicated that BAL samples exhibit a sensitivity of 89% (82-93%) and a specificity of 90% (66-98%) [30]. While BAL can effectively identify confounding conditions such as infection or malignancy, ongoing efforts toward standardization of technique and controlled clinical trials are still in progress [7].

The American Thoracic Society underscores that the accurate interpretation of BAL cellular analyses in ILD necessitates comprehensive clinical information. The clinician’s interpretation of the patient, combined with various diagnostic measures, including BAL, plays a crucial role in the differential diagnosis of these disorders [9]. While BAL remains a valuable tool in the diagnostic evaluation of ILD patients, it is crucial to be mindful of its limitations. Ongoing initiatives to standardize its technique and protocols for processing and analyzing BAL fluid are integral to ensuring its efficacy and reliability in clinical practice.

Technical Challenges and Variability

BAL is a minimally invasive procedure utilized to acquire alveolar cell samples for the assessment of ILD. However, this procedure comes with several limitations and technical challenges, encompassing variations in specimen collection and handling. The recovery rate of lavage fluid can significantly impact results, and artifacts arising from changes in bronchial epithelia may influence the interpretation of the sample. The procedure’s limitations are further compounded by variations in how the specimen is obtained and handled [31]. Despite these challenges, BAL retains its value as a diagnostic tool, with a systematic review and meta-analysis indicating a sensitivity of 89% and a specificity of 90% for BAL samples in lung disease evaluation [30]. It is imperative to recognize these limitations and adhere to recommended techniques for specimen collection and processing to maximize the diagnostic potential of BAL in ILD [30].

Interpretation Challenges and the Need for Expert Analysis

Interpretation challenges: The interpretation of BAL fluid analysis in ILD presents a complex task that demands expert analysis. The clinician’s ability to differentially diagnose ILD relies heavily on their interpretation of the patient’s condition, amalgamated with various diagnostic measures, including the insights derived from BAL [5].

Tissue adequacy: A notable challenge associated with BAL is the potential inadequacy or non-diagnostic nature of the retrieved tissue, posing limitations in certain cases. The variability in tissue quality can impact the overall utility of BAL as a diagnostic tool [9].

Complications: While BAL is generally considered a safe procedure, it is not entirely without risk. Complications such as pyrexia (fever), bronchospasm, and bleeding, although infrequent, can occur. These complications need to be acknowledged and managed to ensure the overall safety of the procedure [20].

Standardization and protocols: The ongoing necessity for the standardization of techniques and protocols for processing and analyzing BAL fluid is a critical aspect. This standardization is essential to guarantee the reliability and consistency of BAL in diagnosing ILD. Establishing uniform practices will contribute to the procedure’s effectiveness and reproducibility across different clinical settings [6].

Future directions and emerging technologies

Advances in BAL Techniques

Flexible bronchoscope: The advent of flexible bronchoscopes in 1966 represents a transformative development that has significantly enhanced the safety and tolerability of BAL. This technological breakthrough allows for precise and targeted sampling of the lower respiratory tract, significantly reducing microbial contamination when compared to conventional sputum analysis. The flexibility and maneuverability of these bronchoscopes contribute to a more controlled and minimally invasive BAL procedure, making it a safer and more well-tolerated diagnostic tool [32].

Optimization of BAL volume and cell yield: A key focus of research in BAL has been the optimization of both the volume of lavage fluid and the resultant cell yield. This research-driven approach aims to enhance the diagnostic accuracy of the procedure. An optimal yield from BAL is considered to be greater than 30% of the instilled volume, translating to more than 70 ml from a 240 ml BAL. This emphasis on achieving an optimal cell yield is crucial for obtaining meaningful diagnostic information, ensuring that the procedure provides valuable insights into the underlying lung pathology [33].

Development of new diagnostic assays: The integration of novel diagnostic assays represents a significant advancement that has fortified the utility of BAL in the context of various lung diseases. These diagnostic assays contribute valuable information for both diagnosis and treatment planning. The incorporation of innovative techniques and assays enhances the diagnostic potential of BAL, allowing clinicians to glean a more nuanced and comprehensive understanding of the cellular and molecular aspects of lung diseases. This development has expanded the scope of BAL’s diagnostic capabilities, making it an increasingly valuable tool in the realm of pulmonary medicine [32].

Standardization of techniques: Achieving uniformity in BAL techniques and the development of standardized protocols for processing and analyzing BAL fluid constitute critical aspects of ongoing research. The standardization of these techniques is paramount for ensuring the reliability and consistency of BAL as a diagnostic tool. By establishing standardized procedures, researchers and clinicians aim to enhance the reproducibility of results, fostering confidence in the diagnostic value of BAL in the evaluation of ILDs [7].

Integration with other diagnostic methods: The integration of BAL with complementary diagnostic methods, such as HRCT, stands as a strategic approach to bolster the accuracy of ILD diagnosis. This collaborative integration has yielded a more comprehensive understanding of the cellular and molecular findings in various lung diseases. By combining the strengths of BAL with other advanced diagnostic techniques, such as imaging modalities, clinicians can obtain a more holistic view of the pathological processes in the lungs. These collaborative efforts contribute to refining the diagnostic accuracy and efficacy of BAL in the broader context of pulmonary medicine [32]. The continuous refinement and integration of BAL techniques with other diagnostic modalities exemplify a multifaceted approach aimed at improving the safety, efficiency, and diagnostic precision of the procedure, underscoring its evolving role in the diagnostic landscape [5,7,32,33].

Integration of Molecular and Genetic Analysis in BAL

Genomic profiling: The application of genomic profiling represents a sophisticated approach wherein BAL samples are subjected to analysis for cancer-associated mutations, specifically in the context of lung cancer diagnosis. This cutting-edge methodology has demonstrated efficacy in the detection of tumors and the identification of mutations that are indicative of various types of lung cancer. The ability to unravel the genomic landscape of lung tumors through BAL samples provides clinicians with valuable insights for precise diagnosis and targeted treatment strategies, emphasizing the potential of genomic profiling as a powerful diagnostic tool [34].

Gene expression analysis: Utilizing BAL cell gene expression analysis has proven instrumental in identifying distinctive gene signatures associated with severe asthma. This analytical approach delves into the intricate molecular mechanisms underlying the disease, revealing new targets for therapeutic intervention. The insights gained from gene expression analysis in BAL cells contribute to a more nuanced understanding of severe asthma, paving the way for the development of tailored therapeutic approaches that address the specific genetic determinants of the condition [35].

Targeted sequencing: In the realm of advanced non-small cell lung cancer, targeted sequencing emerges as a pivotal technique wherein both BAL and tissue samples are subjected to detailed analysis to identify gene variations and assess their performance characteristics. This approach holds promise for uncovering specific genetic aberrations associated with lung cancer and providing crucial information for treatment planning and targeted therapies. By employing targeted sequencing on BAL samples, clinicians can gain a comprehensive understanding of the genetic landscape of lung cancer, offering valuable insights for personalized and precision medicine in the context of this complex disease [36]. The integration of these advanced molecular techniques into BAL analysis showcases a transformative shift toward precision medicine, harnessing the power of genomics to enhance diagnostic accuracy and tailor therapeutic strategies for lung diseases.

Transcriptomics: Delving into the transcriptomics of BAL cells provides a powerful avenue for identifying novel molecular endotypes associated with sarcoidosis. Sarcoidosis, characterized by immune cell accumulation in the lungs, can be better understood through the analysis of gene expression patterns in BAL cells. This transcriptomic approach not only enhances our comprehension of the disease but also holds promise for refining diagnostic methods and advancing treatment strategies. The insights gleaned from transcriptomics offer a molecular-level understanding of sarcoidosis, opening avenues for more targeted and effective interventions [37].

Fiberoptic bronchoscopy with BAL (FOB-BAL): The integration of FOB-BAL represents a dynamic combination that, when coupled with molecular analysis, has demonstrated significant clinical impact. This approach, which involves the use of fiberoptic bronchoscopy to guide BAL, holds the potential to revolutionize the diagnosis and management of various lung diseases. By incorporating molecular analyses, FOB-BAL not only aids in accurate disease identification but also contributes to a more comprehensive understanding of the molecular underpinnings of lung disorders. This innovative approach showcases promise in refining clinical procedures and paving the way for more personalized and effective treatment strategies in the realm of respiratory medicine [38].

Potential Role of Artificial Intelligence (AI) in BAL Interpretation

Anatomical localization with AI: Recent advancements in the application of AI have been directed toward the interpretation of BAL fluid samples to enhance the diagnosis of ILD. A noteworthy study in this domain introduced an AI model designed to accurately identify and distinguish anatomical locations based on bronchoscopic images. This technological innovation holds the potential to precisely pinpoint the areas sampled during BAL procedures, contributing to increased precision and reliability in diagnostic assessments [39].

Automated cytological background analysis: Another significant stride in the integration of AI with BAL involves the development of a model capable of automatically providing the cytological background of BAL fluid. This novel approach aims to streamline and expedite the analysis of cellular components in BAL, offering automation in the identification and categorization of cells present in the fluid. By leveraging AI for cytological background analysis, Tao et al.'s study represents a step forward in enhancing the efficiency and accuracy of BAL interpretation for ILD diagnosis [40]. Despite these promising developments, it is crucial to note that the standardization of BAL techniques and protocols for processing and analyzing BAL fluid is an ongoing endeavor. The clinical utility of AI in BAL interpretation for ILD diagnosis, while showing potential, has not yet been fully established. The integration of AI into the diagnostic landscape of ILD through BAL interpretation awaits further validation and refinement as the field progresses [5,7].

## Conclusions

In conclusion, the comprehensive review underscores the diagnostic precision of BAL in diverse ILDs. The analysis reveals the significance of abnormalities in BAL fluid’s cellular and non-cellular components, emphasizing its potential as a valuable diagnostic tool. Mainly, BAL demonstrates a differentiated diagnostic value in specific ILDs, such as IPF, sarcoidosis, HP, and CTD-ILD. To optimize ILD diagnosis, recommendations include the incorporation of BAL in clinical guidelines, advocating for a multidisciplinary approach involving pulmonologists, radiologists, and pathologists, and encouraging further standardization of BAL procedures. Ongoing research suggests promising avenues, including technological advancements in BAL, biomarker discovery in BAL fluid, and the integration of AI for enhanced data interpretation. These developments not only hold the potential for refining ILD diagnosis but also pave the way for a more personalized approach to patient management through insightful patient stratification based on BAL findings.

## References

[1] Antoine MH, Mlika M (2023). Interstitial lung disease. StatPearls [Internet].

[2] Cottin V, Hirani NA, Hotchkin DL (2018). Presentation, diagnosis and clinical course of the spectrum of progressive-fibrosing interstitial lung diseases. Eur Respir Rev.

[3] Yioe V, Phillips G, Spencer LG (2021). Interstitial lung disease on the acute take for the non-respiratory physician. Clin Med (Lond).

[4] Stanzel F (2012). Bronchoalveolar lavage. Principles and Practice of Interventional Pulmonology.

[5] (2024). Role of Bronchoalveolar Lavage in Diagnosis of Interstitial Lung Disease. https://www.uptodate.com/contents/role-of-bronchoalveolar-lavage-in-diagnosis-of-interstitial-lung-disease.

[6] Patel PH, Antoine MH, Ullah S (2023). Bronchoalveolar lavage. StatPearls [Internet].

[7] Meyer KC, Raghu G (2011). Bronchoalveolar lavage for the evaluation of interstitial lung disease: is it clinically useful?. Eur Respir J.

[8] Tzilas V, Digalaki A, Bouros E, Avdoula E, Tzouvelekis A, Bouros D (2023). Diagnostic utility of bronchoalveolar lavage lymphocytosis in patients with interstitial lung diseases. Respiration.

[9] Meyer KC, Raghu G, Baughman RP (2012). An official American Thoracic Society clinical practice guideline: the clinical utility of bronchoalveolar lavage cellular analysis in interstitial lung disease. Am J Respir Crit Care Med.

[10] Efared B, Ebang-Atsame G, Rabiou S (2017). The diagnostic value of the bronchoalveolar lavage in interstitial lung diseases. J Negat Results Biomed.

[11] (2024). Bronchoscopy and Bronchoalveolar Lavage (BAL). https://medlineplus.gov/lab-tests/bronchoscopy-and-bronchoalveolar-lavage-bal/.

[12] Labmate I (2024). How Are BAL Samples Processed?. https://www.labmate-online.com/news/news-and-views/5/breaking-news/how-are-bal-samples-processed/56327.

[13] Chang SL, Tsai HC, Lin FC, Chao HS, Chou CW, Chang SC (2020). Clinical usefulness of bronchoalveolar lavage in patients with interstitial lung diseases: a pilot study. J Thorac Dis.

[14] Clark BD, Vezza PR, Copeland C, Wilder AM, Abati A (2002). Diagnostic sensitivity of bronchoalveolar lavage versus lung fine needle aspirate. Mod Pathol.

[15] Khan WB, Gallagher HM, Jayasimhan D, Dray M, Chang CL (2023). The impact of bronchoalveolar lavage on the diagnosis of undifferentiated interstitial lung disease alongside a multidisciplinary discussion. Chron Respir Dis.

[16] Harbeck RJ (1998). Immunophenotyping of bronchoalveolar lavage lymphocytes. Clin Diagn Lab Immunol.

[17] Heron M, Grutters JC, ten Dam-Molenkamp KM (2012). Bronchoalveolar lavage cell pattern from healthy human lung. Clin Exp Immunol.

[18] Midulla F, Strappini P, Sandstrom T (2001). Cellular and noncellular components of bronchoalveolar lavage fluid in HIV-1-infected children with radiological evidence of interstitial lung damage. Pediatr Pulmonol.

[19] Van Hoecke L, Job ER, Saelens X, Roose K (2017). Bronchoalveolar lavage of murine lungs to analyze inflammatory cell infiltration. J Vis Exp.

[20] Tanriverdi H, Erboy F, Altinsoy B (2015). Bronchoalveolar lavage fluid characteristics of patients with sarcoidosis and nonsarcoidosis interstitial lung diseases: ten-year experience of a single center in Turkey. Iran Red Crescent Med J.

[21] Kono M, Miyashita K, Hirama R (2021). Prognostic significance of bronchoalveolar lavage cellular analysis in patients with acute exacerbation of interstitial lung disease. Respir Med.

[22] (2024). Idiopathic Pulmonary Fibrosis. https://www.nhs.uk/conditions/idiopathic-pulmonary-fibrosis/.

[23] Association AL (2024). Idiopathic Pulmonary Fibrosis (IPF). https://www.lung.org/lung-health-diseases/lung-disease-lookup/idiopathic-pulmonary-fibrosis.

[24] Watson S, Seed S (2024). Idiopathic Pulmonary Fibrosis (IPF). https://www.webmd.com/lung/what-is-idiopathic-pulmonary-fibrosis.

[25] (2024). Idiopathic Pulmonary Fibrosis. https://www.pulmonaryfibrosis.org/understanding-pff/types-of-pulmonary-fibrosis/idiopathic-pulmonary-fibrosis.

[26] Winterbauer RH, Lammert J, Selland M, Wu R, Corley D, Springmeyer SC (1993). Bronchoalveolar lavage cell populations in the diagnosis of sarcoidosis. Chest.

[27] Bonella F, Costabel U (2020). The perpetual enigma of bronchoalveolar lavage fluid lymphocytosis in chronic hypersensitivity pneumonitis: is it of diagnostic value?. Eur Respir J.

[28] Mlika M, Ben Kilani M, Berraies A, Braham E, Hamzaoui A, Mezni F (2016). Diagnostic value of the bronchoalveolar lavage in interstitial lung disease. Tunis Med.

[29] Costa E Silva M, Rolo R (2017). The role of bronchoalveolar lavage in interstitial lung diseases. Rev Port Pneumol (2006).

[30] Guo Q, Xiao Y, Zhang S (2024). Metagenomic next generation sequencing of bronchoalveolar lavage samples for the diagnosis of lower respiratory tract infections: a systematic review and meta-analysis. Heliyon.

[31] Shen Y, Pang C, Wu Y (2016). Diagnostic performance of bronchoalveolar lavage fluid CD4/CD8 ratio for sarcoidosis: a meta-analysis. EBioMedicine.

[32] Davidson KR, Ha DM, Schwarz MI, Chan ED (2020). Bronchoalveolar lavage as a diagnostic procedure: a review of known cellular and molecular findings in various lung diseases. J Thorac Dis.

[33] Shaw JA, Meiring M, Allies D (2023). Optimising the yield from bronchoalveolar lavage on human participants in infectious disease immunology research. Sci Rep.

[34] Nair VS, Hui AB, Chabon JJ (2022). Genomic profiling of bronchoalveolar lavage fluid in lung cancer. Cancer Res.

[35] Weathington N, O'Brien ME, Radder J (2019). BAL cell gene expression in severe asthma reveals mechanisms of severe disease and influences of medications. Am J Respir Crit Care Med.

[36] Lin X, Cai Y, Zong C (2023). Bronchoalveolar lavage as potential diagnostic specimens to genetic testing in advanced nonsmall cell lung cancer. Technol Cancer Res Treat.

[37] Vukmirovic M, Yan X, Gibson KF (2021). Transcriptomics of bronchoalveolar lavage cells identifies new molecular endotypes of sarcoidosis. Eur Respir J.

[38] Oren I, Hardak E, Zuckerman T, Geffen Y, Hoffman R, Yigla M, Avivi I (2016). Does molecular analysis increase the efficacy of bronchoalveolar lavage in the diagnosis and management of respiratory infections in hemato-oncological patients?. Int J Infect Dis.

[39] Tsai YM, Kuo YS, Lin KH, Chen YY, Huang TW (2023). Diagnostic performance of electromagnetic navigation versus virtual navigation bronchoscopy-guided biopsy for pulmonary lesions in a single institution: potential role of artificial intelligence for navigation planning. Diagnostics (Basel).

[40] Tao Y, Cai Y, Fu H, Song L, Xie L, Wang K (2022). Automated interpretation and analysis of bronchoalveolar lavage fluid. Int J Med Inform.

